# A high-strength mineralized collagen bone scaffold for large-sized cranial bone defect repair in sheep

**DOI:** 10.1093/rb/rby020

**Published:** 2018-08-13

**Authors:** Shuo Wang, Zhijun Zhao, Yongdong Yang, Antonios G Mikos, Zhiye Qiu, Tianxi Song, Fuzhai Cui, Xiumei Wang, Chunyang Zhang

**Affiliations:** 1 State Key Laboratory of New Ceramics and Fine Processing, Key Laboratory of Advanced Materials of Ministry of Education, School of Materials Science and Engineering, Tsinghua University, Beijing, China; 2 Department of Neurosurgery, The First Affiliated Hospital of Baotou Medical School, Baotou, China; 3 Dongzhimen Hospital Affiliated Beijing University of Chinese Medicine, Beijing, China; 4 Department of Bioengineering, Rice University, Houston, TX, USA; 5 Beijing Allgens Medical Science and Technology Co., Ltd., Beijing, China

**Keywords:** mineralized collagen, cranial bone defect, bone regeneration, sheep, cranioplasty materials

## Abstract

Large-sized cranial bone defect repair presents a great challenge in the clinic. The ideal cranioplasty materials to realize the functional and cosmetic recovery of the defect must have sufficient mechanical support, excellent biocompatibility, good osseointegration and biodegradability as well. In this study, a high-strength mineralized collagen (MC) bone scaffold was developed with biomimetic composition, microstructure and mechanical properties for the repair of sheep large-sized cranial bone defects in comparison with two traditional cranioplasty materials, polymethyl methacrylate and titanium mesh. The compact MC scaffold showed no distinct pore structure and therefore possessed good mechanical properties. The strength and elastic modulus of the scaffold were much higher than those of natural cancellous bone and slightly lower than those of natural compact bone. *In vitro* cytocompatibility evaluation revealed that the human bone marrow mesenchymal stem cells (hBMSC) had good viability, attachment and proliferation on the compact MC scaffold indicating its excellent biocompatibility. An adult sheep cranial bone defect model was constructed to evaluate the performances of these cranioplasty materials in repairing the cranial bone defects. The results were investigated by gross observation, computed tomography scanning as well as histological assessments. The *in vivo* evaluations indicated that compact MC scaffold showed notable osteoconductivity and osseointegration with surrounding cranial bone tissues by promoting bone regeneration. Our results suggested that the compact MC scaffold has a promising potential for large-sized cranial bone defect repair.

## Introduction

Cranial bone defects present a common clinical problem and could be caused by congenital defects of dysraphism and skeletal anomalies, or acquired injuries from trauma, encephalic and maxillofacial surgeries and infection. As a consequence, it can trigger multiple physiological complications as well as a negative influence on psychology [1–3]. A patient with a cranial bone defect may suffer from infection, brain swelling, hydrocephalus, epilepsy or hemiplegia, which pose extremely high risk to life. Moreover, relevant psychological and social problems happen quite often due to the patient’s unusual appearance [4, 5]. The common treatment method of cranial bone defects in the clinic is a surgical intervention to reconstruct the skull with materials that provide both stable biomechanical support in function and optimal cosmetic results in appearance, which is called cranioplasty [6].

Biomaterials play an important role in cranioplasty as biocompatible implants that supplement the loss of natural bone, especially for large-sized cranial bone defect repair. A wide range of materials have currently been adopted in cranioplasty, including many kinds of artificial substitutes and natural bone [7, 8]. Closing a large-sized skull defect with full contour matching and complete coverage using autologous bone grafts from the tibia, rib, scapula or ilium is quite difficult to achieve, although it has excellent osteoconductivity and osteoinductivity as the widely recognized ‘gold standard’ of bone repair. Therefore, a variety of biomaterials including bioglass, titanium (Ti), poly(etheretherketone), poly(methyl methacrylate) (PMMA) and hydroxyapatite have been developed for cranioplasty. However, none of these conventional biomaterials are satisfying. For example, Ti, the most commonly used cranioplasty material, may produce significant image artifacts in computed tomography (CT) and magnetic resonance imaging, and can damage brain tissue due to its heat conduction [5, 9]. Besides, the mechanical properties of Ti are excessively higher than that of a human’s natural bone, leading to serious distortion or atrophy of the calvaria several years after implantation. For PMMA, as another example, although its mechanical properties are more comparable to natural bone tissue, it is too brittle to keep its integrity *in vivo* for long-term usage. Furthermore, it is noted that most of the biomaterials in use in the clinic are bioinert or non-biodegradable materials, implying that these materials could neither induce bone regeneration nor be replaced by the nascent bone tissue; thus, the osseointegration between the cranioplasty materials and the surrounding cranial bones is poor. Currently, many researchers studying cranioplasty concentrate on the development of novel bone materials to induce cranial bone regeneration [10–12]. Those typical porous bone scaffolds that were successfully used in bone regeneration showed good osteoconductivity to promote bone regeneration, but their deficient bending strength and toughness limited their applications in broad cranial bone repair. Therefore, achieving adequate bone regeneration for repairing large-sized cranial bone defects remains a great challenge.

Bone scaffold based on mineralized collagen (MC) has been widely used as a regenerative biomaterial for long bone repair and spinal fusion reconstruction both in research and in the clinic for a long time, and it is fabricated via an *in vitro* biomimetic mineralization process to simulate not only the composition, but also the hierarchically self-assembled organization of natural bone tissue [13–18]. It has been proven that the porous scaffolds based on MC show excellent osteogenic capability and osteoconductivity to promote osteogenic differentiation of mesenchymal stem cells *in vitro* and bone regeneration *in vivo* [19–23]. Nevertheless, the mechanical properties of the porous MC scaffolds were not high enough to provide sufficient strength for weight-bearing bone regeneration. Therefore, a high-strength MC-based bone scaffold with compact structure and appropriate mechanical properties was fabricated for large-sized cranial bone defect repair. In this study, the physicochemical properties and *in vitro* biocompatibility of the compact MC scaffold (*c*MC) was evaluated. We then created a large-sized cranial bone defect model in adult sheep to compare the performances of *c*MC with PMMA and titanium mesh in repairing the cranial bone defect.

## Materials and methods

### Preparation of the MC-based bone scaffold

The MC powder was prepared as reported previously [13]. Briefly, the Ca^2+^ and ${\text{PO}}_{4}^{3-}$ ions were dropped into acidic type I collagen solution. The pH value of the prepared solution was adjusted to 7.4 with constant stirring for 48 h. Then the MC deposition was gradually formed and harvested by centrifugation, lyophilized and then ground into powder for use. *c*MC, the MC-based scaffold with high mechanical properties, consisted of poly(ε-caprolactone) (PCL) and MC. PCL (Jinan Daigang Biomaterial Co., biomedical grade, 300 kDa) was mixed with MC powder homogeneously with a weight ratio of 1:1 and then molded into a disc shape of 30 mm in diameter and 3 mm in height by mechanical force into a mold. The scaffolds were sterilized by ^60^Co irradiation and then stored in a sterilized state until use.

### Characterization of the physicochemical properties of the scaffolds

The microstructure of the *c*MC scaffold was observed by field emission scanning electron microscopy (SEM, Merlin Zeiss, Germany), involving the outer surface of as-prepared *c*MC scaffold as well as the fresh fractured surface. The samples were fixed on a specimen stage using a conductive tape and then coated with a layer of gold film.

The compressive strength and the elastic modulus of the scaffolds were measured using a universal mechanical testing machine (SHIMADZU AG-IC, Japan). The shape of samples for testing was in accordance with the standard cylinders, 20 mm in length and 10 mm in diameter. A 250 N load cell was set on the cylinder scaffolds to provide a force from 0 N and the force continued to increase until the scaffold experienced a deformation of 30% vertically. The slope of the initial linear portion of the stress–strain curve was considered as the elastic modulus. A line with the same slope of the elastic modulus was drawn from the 20% strain point to reach an intersection with the curve, which was regarded as the compressive strength. Three individual standard samples of *c*MC scaffold were measured repeatedly for statistical analysis.

### *In vitro* cytocompatibility of the scaffolds

hBMSC (passage 6–8, Cyagen Biosciences Inc.) were cultured in glucose Dulbecco’s modified eagle medium, in which the percentage of fetal bovine serum and penicillin-streptomycin solution were 10% and 1%, respectively. The cells were cultured in an incubator under an atmosphere of 5% CO_2_ at 37°C.

The cells’ adhesion state on the *c*MC scaffold was examined by SEM. The concentration of cells seeded on the three different materials (*c*MC, PMMA and Ti) in six-well cell culture plates was 2 × 10^5^ cells/well. Then the samples were taken out and fixed with 2.5% glutaraldehyde in phosphate buffered saline (0.1 M, pH = 7.4) after 24-h regular culture, followed by gradient dehydration up to 100% ethanol. The prepared cell-containing scaffold was finally dried through critical point drying (Samdri-PVT-3D, America), and then coated with a layer of gold film for observation by SEM. Cell proliferation was then measured via Cell Counting Kit-8 (CCK-8, Dojindo, Japan). The hBMSCs were seeded on the samples in 6-well cell culture plates with a concentration of 1 × 10^5^ per well and examined at 1, 4, or 7 d after cell seeding. All the measurements at each time point were repeated three times [24, 25].

### *In vitro* osteogenic differentiation of stem cells on the *c*MC scaffold

The *in vitro* osteogenic capacity of the MC has been proved in our previous works [21, 26]. To confirm the osteogenic property of *c*MC, reverse transcription polymerase chain reaction (RT-PCR) was used to test the expression of osteogenesis-related genes. Sprague Dawley rat bone mesenchymal stem cells (SD-rat BMSCs) were seeded onto the *c*MC scaffold at a concentration of 1.5 × 10^5^ cells per well in 6-well cell culture plates. Round coverslips with the same area were set as a control. When the cells grew to about 70% confluence, the medium was changed to osteogenic induction medium (RASMX-90021, Cyagen Biosciences Inc.) and the cells were cultured for another two weeks. Then the total cellular messenger ribonucleic acid (mRNA) was isolated and purified via miRcute miRNA Isolation Kit (DP501, TIANGEN Biotech Co., Ltd.), and the complementary deoxyribonucleic acid was obtained using FastQuant RT Kit (KR106, TIANGEN Biotech Co., Ltd.). RT-PCR was performed using iTaq Universal SYBR Green Supermix (172-5121, BIO-RAD) via Thermal Cycler (T100, BIO-RAD) and the relative level of gene expressions including ALP, Runx2, BMP-2, OPN and Col 1 of SD-rat BMSCs (passage 4–6, RASMX-01001, Cyagen Biosciences Inc.) were measured by Real-Time System (CFX96, BIO-RAD). The data were recorded and then calculated using the 2^−^^ΔΔCt^ method. The primer sequences (Beijing Genomics Institute, BGI, China) were designed by referring to some similar works related to SD-rat BMSCs’ osteogenic differentiation [10, 27, 28]. The primer sequences are shown in Table 1.

**Table 1:** Primer sequences used for RT-PCR gene expression analysis

Genes	5′-3′	Primers	Production size (bp)
ALP	Forward	CCTGGACCTCATCAGCATTT	279
	Reverse	AGGGAAGGGTCAGTCAGGTT	
Runx2	Forward	TCTCTGACCGCCTCAGTGATT	171
	Reverse	TGTGTCTGCCTGGGATCTGTA	
BMP-2	Forward	GAAGCCAGGTGTCTCCAAGAG	142
	Reverse	GTGGATGTCCTTTACCGTCGT	
OPN	Forward	GGAGTCCGATGAGGCTATCAA	208
	Reverse	TCCGACTGCTCAGTGCTCTC	
Col 1	Forward	TGGATGGCTGCACGAGT	177
	Reverse	TTGGGATGGAGGGAGTTTA	

### *In vivo* evaluation of *c*MC for cranial bone repair in sheep model

A large-sized cranial bone defect (30 mm in diameter) was created in healthy 1-year-old sheep to construct an animal model for evaluating different implants. In total, 16 healthy 1-year-old sheep were randomly divided into four groups for four various repair treatments: no implant (blank group), MC-based composite scaffold implant (*c*MC group), Ti mesh implant (Ti-mesh group) and PMMA implant (PMMA group). All surgeries were carried out at the First Affiliated Hospital of Baotou Medical College, China.

**Figure 1:**
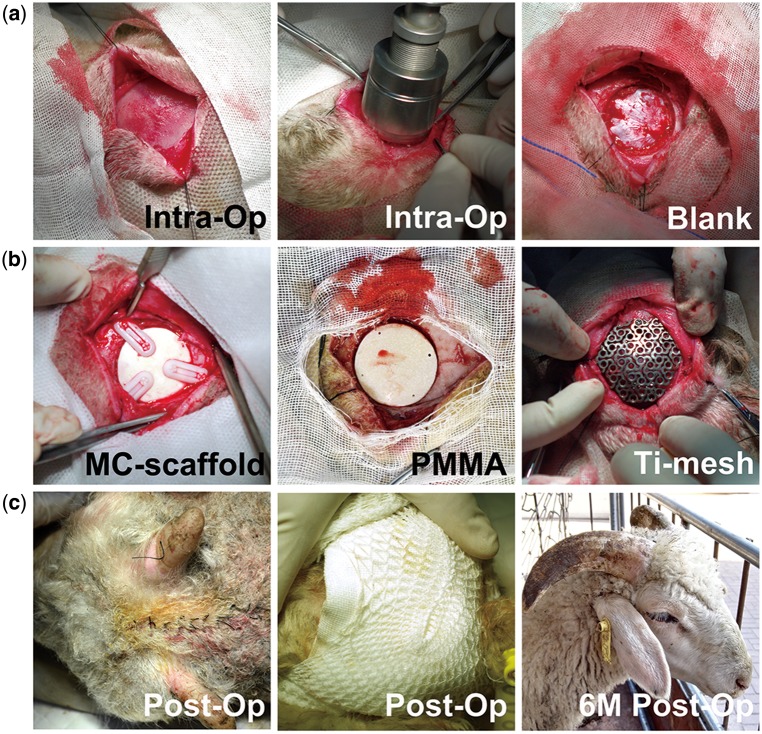
Surgical procedures of skull reconstruction in a 1-year-old sheep cranial bone defect model. (**a**) Construction of 3 cm cranial bone defects. (**b**) Implantation of different bone materials into the defects. (**c**) Observation of sheep’s appearances immediately after surgery and 6 months after surgery

After intravenous injection of 3% sodium pentobarbital (30 mg/kg weight), the sheep were shaved and incisions in the skin were made at the position of calvariae to expose the cranial bone, partially destroying the periosteum on the cranial bone. The defect was created by rongeur forceps after locating the center of the calvaria of each sheep with a bone drill, leaving a 30-mm diameter round defect with intact dura mater (Fig. 1a). Then, different kinds of implant were placed into the defects (Fig. 1b) and the wounds were sutured carefully, making sure that the implants were at proper positions (Fig. 1c). A total of 1600 000 IU penicillin was given through an intramuscular route once a day for 5 days after the surgeries.

### CT imaging

CT scans of the sheep heads were performed to detect the status of cranial bone defect regeneration immediately post-surgery as well as 6 months after surgery. Both the X-ray scan images and the three-dimensional (3D) reconstruction images were obtained to compare the effects of different implants and the repair outcomes.

### Histological assessments

Six months after the operation, all the animals were euthanized. The cranial bones including the implant or defect area and the surrounding original cranial bone were harvested carefully and immediately fixed with 4% formaldehyde for 48 h. After gradient dehydration and hyalinization, the tissue blocks were embedded in a mixture of methyl methacrylate, dibutyl phthalate and benzoperoxide and solidified at 40°C. After that, the embedded tissue blocks were cut into 5-μm thick slices by hard tissue slicing (LEICA, 2500E, Germany) and stained by Masson’s trichrome staining as well as Hematoxylin-Eosin (H&E) staining. The details of the stained slices were observed by an automatic digital slide scanning system (Zeiss, Axio Scan Z1, Germany).

### Statistical analysis

The numerical data were reported in the form of mean ± standard deviation. The data were considered statistically significant with a *P*-value < 0.05 via one-way ANOVA. The data were analyzed by Minitab 17 software for Windows.

## Results

### The physicochemical properties of the *c*MC composite scaffold

**Figure 2:**
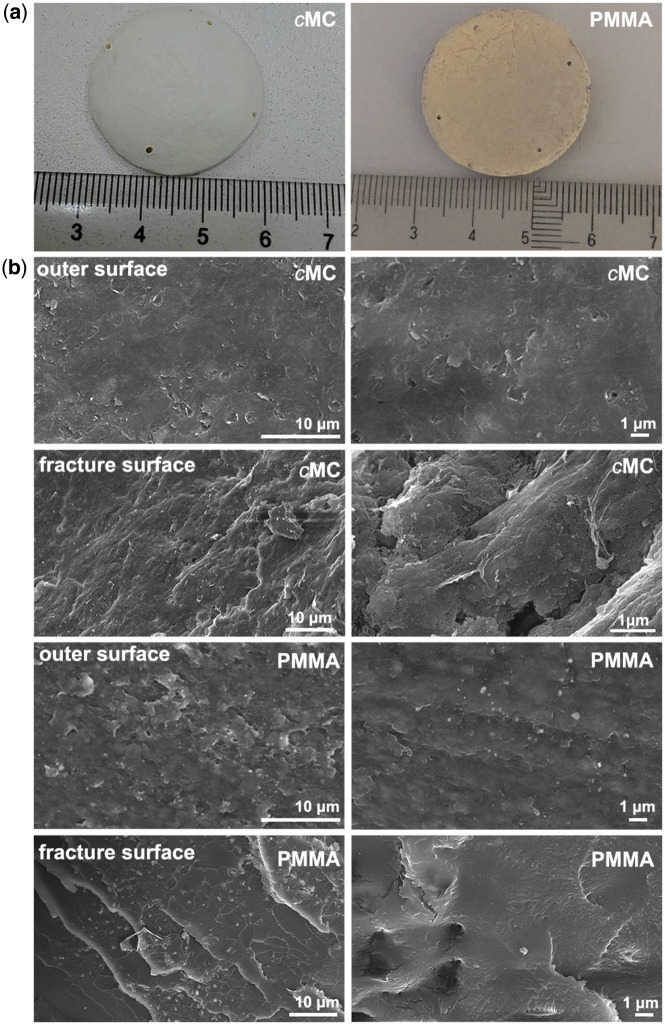
Representative gross and SEM morphologies of MC scaffold and PMMA. (**a**) Macroscopic image of MC scaffold and PMMA. (**b**) SEM images of the outer as well as the fracture surface of MC scaffold and the outer surface of PMMA

The gross profiles of the *c*MC composite and PMMA implants in a 30-mm-diameter disk are shown in Fig. 2a. The *c*MC and PMMA scaffolds appeared homogenous and compact, which was confirmed by the SEM examinations. As shown in Fig. 2b, the representative SEM morphologies of the outer surfaces of the *c*MC and PMMA scaffolds exhibited compact and smooth microstructures and almost no pore could be observed even under high magnification. Moreover, the fracture surface of *c*MC scaffold appeared relatively rugged, indicating the toughness of *c*MC, in contrast to the flat surface of brittle fracture for PMMA.

The mechanical properties of the *c*MC as well as PMMA scaffolds were obtained according to the stress–strain curve from the compression test. The elastic modulus and compressive strength of *c*MC scaffold were 0.60 ± 0.01 GPa and 32.6 ± 0.8 MPa, respectively, versus 1.77 ± 0.16 GPa and 84.65 ± 4.45 MPa for PMMA scaffold. As listed in Table 2, it is noted that the compressive strength values of the *c*MC and the PMMA scaffolds were close to that of natural compact bone, while the elastic modulus and the compressive strength of Ti are about 50–60 GPa and 4–10 × 10^2^ MPa, which are much higher than those of nature bones [29, 30]. What is more, the density of *c*MC scaffold was 1.72 ±0.05 g/cm^3^ which is similar to the density of natural skull bone (1.7–1.8 g/cm^3^).

**Table 2:** Mechanical properties of bone materials and natural bone

	*c*MC scaffold	PMMA	Ti	Cancellous bone	Compact bone
Compressive strength (MPa)	32.6 ± 0.8	84.65 ± 4.45	400–1000	1–10	100–200
Elasticity modulus (GPa)	0.60 ± 0.01	1.77 ± 0.16	50–60	0.1–3	10–20

### *In vitro* cytocompatibility of *c*MC scaffold

**Figure 3:**
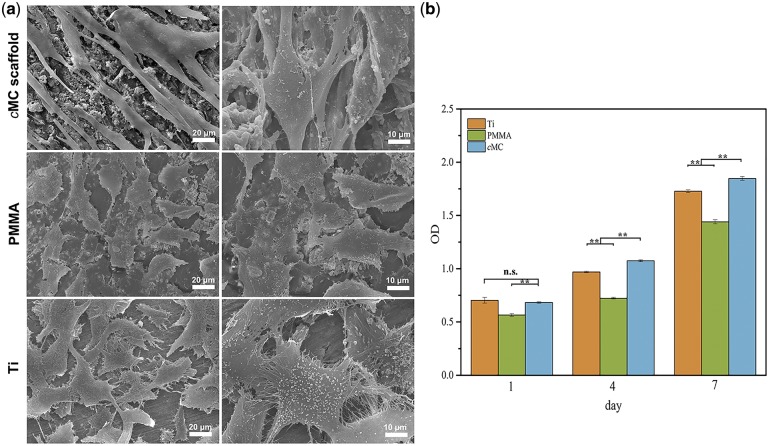
*In vitro* biocompatibility of MC scaffolds. (**a**) SEM images of hBMSCs on the surfaces of MC scaffold, PMMA, and Ti after 12 h of cell culture. (**b**) CCK-8 assay of cell proliferation on MC scaffold, PMMA, and Ti. The data represent the means ± SD. **, *P* < 0.01; ns, no statistical difference

After cell seeding, most of the hBMSCs attached on the scaffolds and maintained viability; no dead cells were observed floating in the culture medium. The SEM morphologies of hBMSCs cultured on *c*MC scaffolds indicated that the cells underwent adhesion and spreading, displaying typical spindle cell shape and protruded pseudopods (Fig. 3a). However, the cells on the surface of PMMA as well as Ti plate showed less adhesion compared with *c*MC, which could be judged by the polygonal cell shape and the lack of obvious protruded pseudopods. The fibrous pseudopods of cells on *c*MC were much longer than that of the other two groups and closely connected to each other. Furthermore, as shown in Fig. 3b, the cell proliferation behaviors measured by CCK-8 revealed that none of the materials were cytotoxic to the hBMSCs and the increase of cell population was obvious during the whole culture period for all groups. It is also worth mentioning that the cells on *c*MC had the greatest viability and the fastest proliferation rate, suggesting that the scaffold possessed excellent cytocompatibility and biological activity.

### *In vitro* osteogenic differentiation of BMSCs on the *c*MC scaffold

**Figure 4:**
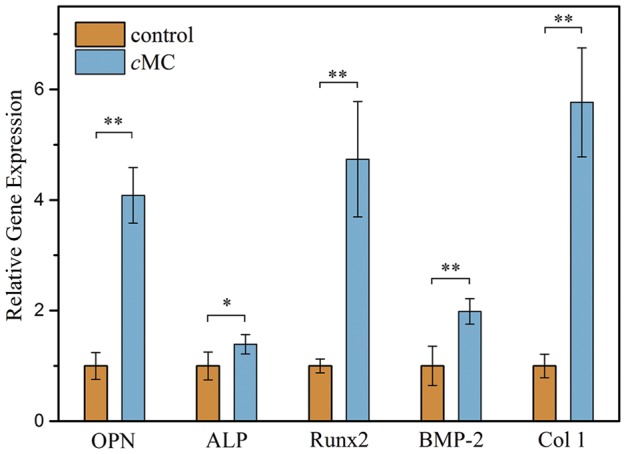
Levels of mRNA for related genes (OPN, ALP, Runx2, BMP-2, and Col 1) in ostogenic differentiation of SDrat BMSCs on *c*MC scaffolds. The data represent the means ± SD. **, *P* < 0.01; *, *P* < 0.05

To examine the activity of the MC component in the *c*MC scaffold on rat BMSCs’ osteogenic differentiation, the relative expression of osteogenesis-associated genes, including ALP, Runx2, BMP-2, OPN and Col 1 were measured via RT-PCR, compared with coverslip control group. As shown in Fig. 4, the expression of related genes in the BMP signaling pathway, Runx2 and BMP-2, were higher than for the control group. BMP-2 expression was increased 2.0-fold (*P* < 0.01) on cMC and Runx2 was remarkably increased 4.7-fold (*P* < 0.01). What is more, the OPN and Col 1 expression levels were increased 4.1-fold and 5.8-fold respectively, revealing a relatively high expression level of osteogenic protein on cMC. The expression of ALP, an early osteogenic marker, was slightly higher than that of the coverslip group, about 1.4-fold (*P* < 0.05).

### *In vivo* cranial bone regeneration

**Figure 5:**
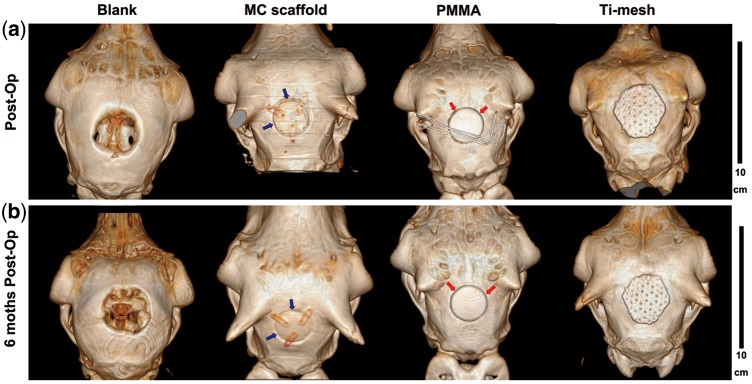
Images of the sheep skulls with different implants. (**a**) CT 3D reconstructed images immediately after operation. (**b**) CT 3D reconstructed images at 6 months post-operation

CT imaging was conducted immediately after surgery and at 6 months post-operation to examine the repair of cranial bone defects by using different scaffolds. The 3D reconstructed CT images clearly exhibited the obvious distinctions among all the groups and the variations after 6 months of recovery, as shown in Fig. 5. In the blank group, there was a distinct 30-mm round empty defect on the calvariae, representing the defect created during the surgery. In the other three groups implanted with different biomaterials, the defects were enclosed completely and repaired well. The three implants, *c*MC, PMMA, and Ti-mesh could be clearly distinguished in the CT images, with similar or higher density relative to the natural cranial bone for blocking the X-ray. In the *c*MC scaffold group as well as the PMMA group, the outlines of the scaffolds were apparent, the dark lines of which revealed the interfaces between the round disk of implants and the surrounding cranial bone (Fig. 5a). Meanwhile, the hexagonal Ti-mesh implant was larger than the defect size in order to fix it on the cranium via screws. At 6 months after surgery (Fig. 5b), the defect size in the blank group had no obvious change with only a small amount of nascent bone regeneration along the defect border, indicating that the repair of the 30-mm defect cannot occur spontaneously in the absence of cranioplasty materials. In other groups, the outlines of the three implants were still clearly identified, indicating that no obvious biodegradation had occurred within 6 months. In addition, the interface between the PMMA implant and surrounding bone tissue appeared as dark as in the early stage of post-operation, implying the density of the interface was still very low without increasing within 6 months (indicated by red arrows). In contrast, the density of the interface between the *c*MC implant and surrounding bone tissue increased in a manner that can probably be attributed to the excellent osseointegration ability of the *c*MC scaffold to promote new bone formation (indicated by blue arrows). The observations for the Ti-mesh group were similar to those for the PMMA group in that no obvious variation happened over time, judging from the 3D reconstructed images.

**Figure 6:**
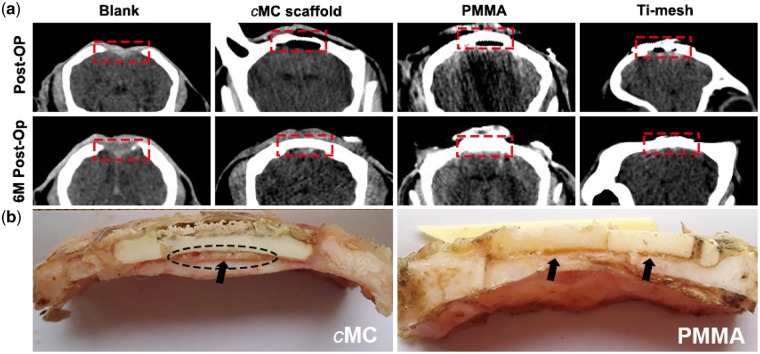
(**a**) X-ray coronal scan images of the sheep skulls with different implants immediately after operation and at 6 months post-operation. (**b**) Gross observation of the isolated cranial bones with *c*MC and PMMA

In order to clearly show the tissue features surrounding the implants, the morphologies of the cross-sections were also examined, as shown in Fig. 6. The X-ray coronal scan images shown in Fig. 6a revealed more details of the regeneration of the defect area (the defect area in each group were marked by a red rectangle). In the blank group, the defect showed no obvious recovery after 6 months, even though there was soft connective tissue regenerated in the lost area (according to gross observation, data not shown), which was not detectable under X-ray beam. In the PMMA and Ti-mesh groups, no distinct variations were observed after 6 months. However, in the *c*MC group, there was a cavity beneath the implant because the thickness of the *c*MC scaffold was smaller than that of the cranial bone. At 6 months after surgery, the cavity disappeared and the density increased remarkably. This result indicated that there should be a layer of new bone regenerated along the inner surface of the implant, which made the implant look thicker. The direct observation of the gross morphologies of the cross-sections of the *c*MC and PMMA implants with surrounding tissues are shown in Fig. 6b. From the freshly exposed cross-section, we could clearly see that there were no fibrous tissues formed capsulizing the implants, illustrating both the PMMA and *c*MC scaffolds had good biocompatibility. Additionally, a newly formed bone-like tissue could be identified beneath the *c*MC scaffold and in the slit between *c*MC and the cranial bone, as denoted by the black dotted circle and arrow, which suggested that the *c*MC scaffold had good osteoconduction to promote new bone formation along the scaffold and therefore had good osseointegration with surrounding bone tissues. It is worth mentioning that the bone-like tissues were not found in the PMMA group. What is more, the surface of the *c*MC scaffold was not as flat as it was at the original stage, revealing a certain degree of biodegradation *in vivo* over 6 months. However, no biodegradation happened in the PMMA group. It was also noted that the PMMA scaffold had broken into three pieces due to its brittleness, which may be harmful to the brain tissue under the implant. And there were obvious gaps between PMMA and fiber tissue marked by black arrows. For the Ti-mesh group, there was only transparent, thin, membrane-like soft tissue covering the defects beneath the implants and no sufficient bone regeneration within the defect areas (data not shown).

**Figure 7:**
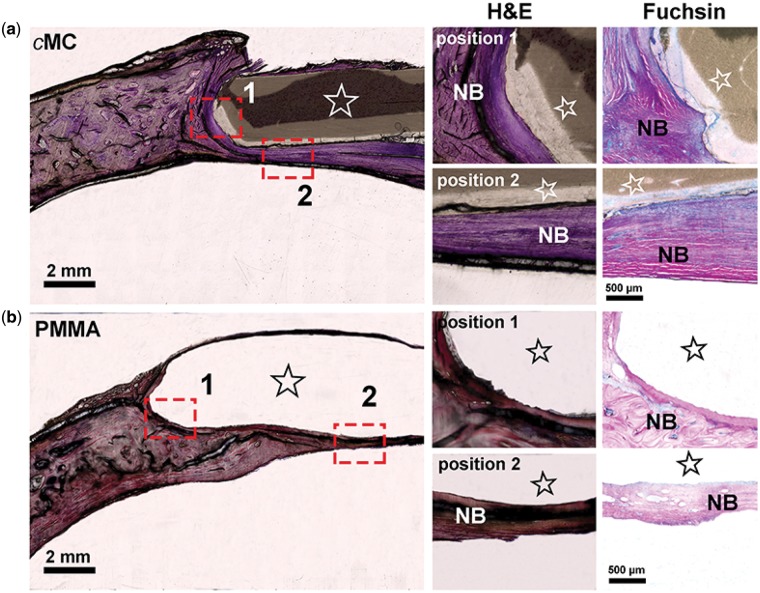
Histological assessment of the cranial bone samples from two kinds of scaffold groups at 6 months post-operation with (**a**) *c*MC and (**b**) PMMA

The histological assessment of *c*MC group as well as PMMA group further exhibited the interfaces between the implants and the surrounding cranial bone tissue, as shown in Fig. 7. The samples underwent hard tissue slicing followed by H&E and Fuchsin staining, the histological examinations of which detected the boundary between tissue and scaffold and confirmed the formation of a bone-like tissue. In both Fig. 7a and b, regions of materials (marked by stars) and tissue could be distinguished in which the *c*MC scaffold was intact and attached quite well with surrounding tissue. However, the PMMA was fragile, and what is more, the bonding with the original peripheral bone was too weak to remain intact during hard tissue slicing with a resulting empty gap, which indicated the bad osseointegration ability of PMMA. The magnified image of **position 1** (marked by the red rectangle) showed no gap between *c*MC and neo-bone (NB), which could be confirmed in Fuchsin staining images by the pink tissue indicating mature bone and the blue tissue indicating immature bone. For the magnified image of **position 2** (marked by the red rectangle) in the *c*MC group, NB formed beneath the implant could be clearly observed, the thickness of which was much higher than for the PMMA group. The PMMA scaffold was mainly surrounded by soft connective tissue. Furthermore, it is noted that the contrast of *c*MC under the optical microscope varied with different regions. The lighter color in the outer layer of the *c*MC indicated the higher light transmission through the *c*MC that was probably attributed to the swelling and partial degradation of the *c*MC scaffold.

## Discussion

Repair of large-sized cranial bone defects by achieving stable biomechanical support and a certain degree of bone regeneration remains a great challenge in the clinic. As a therapy to avoid high intracranial pressure as well as accidental impact, the cranial bone implants should be preferentially designed to satisfy the requirements of the protection of brain tissue. As a result, the biomechanical properties are an essential precondition for the design of cranial bone scaffolds.

Both Ti and PMMA have been widely applied in bone repair especially due to their high strength along with stable chemical properties and good biocompatibility. Though Ti-mesh and PMMA have been widely accepted in the clinic as typical cranioplasty materials, the disadvantages of them cannot be ignored, among which lack of biodegradation and unsatisfactory long-term bone compatibility are two main factors compromising the therapeutic effect. Moreover, there are usually no synostoses between the above two implants and their surrounding original bone in clinical cases. In most research on bone regeneration, porous scaffolds with fast biodegradation within 3 months are frequently utilized. Such porous scaffolds inevitably do not provide adequate mechanical support and especially lack sufficient compressive strength. However, in large-sized cranial bone defect, the area of the defects is usually about dozens of square centimeters or even larger than 100 cm^2^, while the thickness of the cranial bone is about 1–1.5 cm. It is obvious that those typical porous bone materials cannot meet the mechanical requirements of cranioplasty. Therefore, developing a high-strength scaffold with good osseointegration and appropriate osteogenic ability is of vital importance for cranial defect repair in the clinic.

In our previous work, a porous bone scaffold based on MC had been used in a developing cranial bone defect model of sheep [31]. The results showed that the porous MC scaffold could promote bone regeneration and was replaced by the newly formed bone tissue within 3 months, which indicated the excellent osteogenic capability of MC. In this study, in order to meet the mechanical requirements of cranial bone implants for large-sized defect repair, the structure of MC scaffold was designed to be compact with the same composition; as a result, we constructed the *c*MC scaffold with a compressive strength and density close to those of natural compact bone. Though apparent biodegradation was not observed in this study, the long-term degradation behavior is predictable because of the biodegradable components of the scaffold, PCL and MC. Furthermore, a certain degree of swelling and partial degradation at the surface of the *c*MC scaffold were observed and should probably contribute to the osseointegration. According to the gross observation of the freshly exposed cross-sections, a thick layer of bone-like tissue along the inner surface of *c*MC scaffolds was noted without fibrous tissues capsulizing the implant at 6 months post-surgery. X-ray coronal scan images showed no obvious slit between *c*MC and surrounding bone existed, and the implant maintained relative integrity, meaning that *c*MC had excellent osteoconduction and osseointegration properties as well as sufficient compressive strength and toughness to keep its integrity. The synostoses of *c*MC with surrounding bone tissue were confirmed by the histological results, which indicated good bonding force to keep the materials intact during the hard tissue slicing. Instead, the PMMA implants were separated from the surrounding tissue by the shear stress.

Human cranial bones are composed of two thin layers of compact bone enclosing an interposed cancellous bone called diploë, which possess poor self-healing ability and a low regeneration rate in comparison with tubular bones due to the lack of enough blood supply and bone marrow [8, 32–35]. The mechanism of cranial bone regeneration during skull defect repair have been studied, showing that three pathways could be involved in cranial bone regeneration [36–39]. The first pathway of new bone invasion is from the periosteum that is full of osteoblasts/progenitor cells on the outside of natural cranial bone. The second pathway is through the exposed diploë of peripheral cranium. In addition, the outmost periosteal layer of dura mater that serves as the skull’s inner periosteum has similar physiological function to the periosteum for inducing new bone formation as the third pathway [40–44]. In view of the three regeneration pathways, the effect of periosteum could be excluded because the periosteum is usually broken or even absent when a cranial injury arises. At the same time, the nonporous structure of the *c*MC scaffold made the bone regeneration into the scaffold via the diploë layer pathway somewhat difficult before degradation. Nevertheless, a layer of nascent bone quickly formed beneath the *c*MC through the dura mater-derived osteogenesis pathway, and this layer may achieve long-term stable biomechanical support instead of the *c*MC. Thus, the high-strength *c*MC scaffold was able to close the defect and to provide mechanical protection at the initial stage. After that, the newly formed bone tissue beneath the scaffold could also give the mechanical support. Therefore, the slow degradation and softening of *c*MC over a long period will not restrict the application of *c*MC scaffolds in large-sized cranial bone defect repair.

In this work, the *c*MC scaffold as a high-strength cranioplasty material for large-sized cranial bone defect repair showed good biocompatibility, osteoconductivity, and osseointegration effects to form synostoses and a new bone layer. Compared with other materials used in cranial bone regeneration research, such as films, hydrogels and some porous scaffolds, the advantage of mechanical properties of *c*MC is obvious. However, its low biodegradation rate may limit the diploë layer pathway during bone regeneration, and cannot realize fast skull regeneration for some special cases, especially the repair of developing cranial bone defects. Currently, a compact/porous two-phase MC scaffold has been developed and is being evaluated in our laboratory, the compact and porous phases of which were designed to provide enough mechanical support and to quicken the bone regeneration pathway, respectively.

## Conclusions

A new cranioplasty material based on MC with high strength was developed for the repair of large-sized cranial bone defects in sheep. The compact MC scaffold showed no distinct pore structure and therefore possessed good mechanical properties. Additionally, the *c*MC scaffold possessed excellent biocompatibility *in vitro* and *in vivo*. In the adult sheep cranial bone defect model, the *c*MC scaffold showed desirable osteoconductivity and osseointegration with surrounding cranial bone tissues by promoting bone regeneration via the dura mater-derived osteogenesis pathway.
